# *PTEN* and *AKT1* Variations in Childhood T-Cell Acute Lymphoblastic Leukemia

**DOI:** 10.4274/tjh.galenos.2019.2019.0282

**Published:** 2020-05-06

**Authors:** Fulya Küçükcankurt, Yücel Erbilgin, Sinem Fırtına, Özden Hatırnaz Ng, Zeynep Karakaş, Tiraje Celkan, Ayşegül Ünüvar, Uğur Özbek, Müge Sayitoğlu

**Affiliations:** 1 İstanbul University, Aziz Sancar Institute of Experimental Medicine, Department of Genetics, İstanbul, Turkey; 2 Altınbaş University Faculty of Medicine, İstanbul, Turkey; 3 İstinye University Faculty of Art and Science, Department of Molecular Biology and Genetics, İstanbul, Turkey; 4 Acıbadem Mehmet Ali Aydınlar University Faculty of Medicine, Department of Medical Biology, İstanbul, Turkey; 5 İstanbul University Faculty of Medicine, Department of Pediatrics Hematology, İstanbul, Turkey; 6 İstanbul University-Cerrahpaşa Cerrahpaşa Faculty of Medicine, Department of Pediatric Hematology, İstanbul, Turkey; 7 Acıbadem Mehmet Ali Aydınlar University Faculty of Medicine, Department of Medical Genetics, İstanbul, Turkey; α F.K. and Y.E. contributed equally to this work.

**Keywords:** T-ALL, PTEN, AKT1, Next-generation sequencing

## Abstract

**Objective:**

PTEN/AKT pathway deregulations have been reported to be associated with treatment response in acute leukemia. This study examined pediatric T-cell acute lymphoblastic leukemia (T-ALL) samples for *PTEN* and *AKT1* gene variations and evaluated the clinical findings.

**Materials and Methods:**

Fifty diagnostic bone marrow samples of childhood T-ALL cases were investigated for the hotspot regions of the *PTEN* and *AKT1* genes by targeted next-generation sequencing.

**Results:**

A total of five PTEN variations were found in three of the 50 T-ALL cases (6%). Three of the *PTEN* variations were first reported in this study. Furthermore, one patient clearly had two different mutant clones for *PTEN*. Two intronic single-nucleotide variations were found in *AKT1* and none of the patients carried pathogenic *AKT1* variations.

**Conclusion:**

Targeted deep sequencing allowed us to detect both low-level variations and clonal diversity. Low-level *PTEN/AKT1* variation frequency makes it harder to investigate the clinical associations of the variants. On the other hand, characterization of the PTEN/AKT signaling members is important for improving case-specific therapeutic strategies.

## Abstract (tr)

**Amaç:**

PTEN/AKT yolak düzensizliklerinin akut lösemide tedavi yanıtı ile ilişkili olduğu bildirilmiştir. Çalışmanın kapsamı, pediatrik T-ALL hastalarının *PTEN* ve *AKT1* genlerinin sıcak bölge varyasyonları için incelenmesi ve klinik bulgularla değerlendirilmesidir.

**Gereç ve Yöntemler:**

Elli pediatrik T-ALL olgusunun tanı zamanı kemik iliği örnekleri, *PTEN* ve *AKT1* genlerinin sıcak bölgeleri için hedefe yönelik yeni nesil dizileme ile dizilenmiştir.

**Bulgular:**

Elli T-ALL olgusunun %6’sında *PTEN* varyasyonu saptanmıştır. Tespit edilen varyasyonlardan üçü ilk defa bu çalışmada gösterilmiştir. Ayrıca bir hastanın *PTEN* açısından iki farklı mutant klon taşıdığı belirlenmiştir. *AKT1* geninde iki intronik tek nükleotid polimorfizmi tespit edilirken hiçbir olguda patojenik *AKT1* varyasyonu saptanmamıştır.

**Sonuç:**

Derin dizileme, hem düşük düzeydeki varyasyonların hem de klonal çeşitliliğin belirlenmesine olanak sağlamıştır. T-ALL hastalarındaki düşük düzey *PTEN/AKT1* varyasyon sıklığı, varyantların klinikle ilişkisinin ortaya çıkarılmasını zorlaştırmaktadır. Diğer yandan, PTEN/AKT sinyal yolağının karakterizasyonu hasta spesifik terapötik stratejilerin uygulanabilirliği için önemlidir.

## Introduction

One of the key signal transduction pathways involved in malignant transformation is the PTEN/PI3K/AKT pathway, which regulates cellular metabolism, cell growth, translation, chromosome stability, and cell survival [1]. Phosphatase and tensin homolog deleted on chromosome ten (PTEN) is a lipid and dual function phosphatase that antagonizes the PI3K/AKT pathway; by dephosphorylating phosphoinositide 3-kinase (PI3K) it produces PIP2 (phosphatidylinositol 4,5-bisphosphate) and PIP3 (phosphatidylinositol (3,4,5)-triphosphate) and so terminates the transmission of the signal to AKT and other PIP3-effector targets [2]. *AKT1 *is a serine threonine kinase that modulates the cell cycle checkpoint [3]. *AKT1 *is activated by platelet-derived growth factor and its activation is deregulated by mutations in the pleckstrin homology domain of *AKT1*. Survival factors can suppress apoptosis in a transcription-independent manner by activating the serine/threonine kinase *AKT1,* which then phosphorylates and inactivates components of the apoptotic machinery [4].

*PTEN* as a tumor suppressor is frequently mutated in cancers and its inactivation results in constitutive activation of the PI3K/AKT pathway. *PTEN* is a regulatory key to prevent the malignant transformation of T-cells [5]. The PTEN/AKT pathway has an important role in the β-selection checkpoint in T-cell development and lymphocyte homeostasis [6]*. PTEN*-deficient T-cells are found to be highly proliferative as a cause of increased phosphorylation of AKT [7]. *AKT1* is highly expressed in thymus tissue and knockout studies showed that terminal differentiation in CD8+ T-cells failed, with increased proliferation, cytokine secretion, and prolonged survival [8,9]. PTEN/AKT abnormalities resulting in deletion, insertion, or missense mutations lead to differential regulation in different hematologic malignancies [10,11,12,13,14]. Genomic resequencing results showed that PI3K/AKT pathway genes are commonly mutated in pediatric and young adult T-cell acute lymphoblastic leukemia (T-ALL) cases [11,15]. In this study, *PTEN *and *AKT1* variations and their clinical associations were analyzed in a group of childhood T-ALL cases.

## Materials and Methods

Childhood T-ALL cases (n=50) diagnosed at the İstanbul University Faculty of Medicine and Cerrahpaşa University Faculty of Medicine were included in this study. Patients were treated according to the BFM-ALL protocol. Diagnostic bone marrow samples with a blast count of >80% were included in the study. The T-cell origin of ALL was defined by the expression of immunophenotype markers that included CD1a, CD2, cytoplasmic CD3, surface CD3, CD4, CD5, CD7, and CD8. T-ALL cases were evaluated according to the European Group for the Immunological Characterization of Leukemia classification scale as immature (n=20), cortical (n=17), or mature (n=4); however, nine cases were not able to be further classified due to limited immunological marker information [11]. Median age was 8 (range=0.9-17) years and other clinical features of the T-ALL cases are summarized in Table 1. Written and oral informed consent was obtained from the legal representatives of the pediatric patients.

### Identification of *PTEN* and *AKT1* variations

The mononuclear cells of the bone marrow samples were isolated by the Ficoll density gradient procedure [16]. Genomic DNA was isolated with the QIAamp DNA Mini Kit (QIAGEN GmbH, Hilden, Germany) according to the manufacturer’s protocol. DNA quality and quantity were checked with a spectrophotometer (NanoDrop 100, Thermo Scientific, USA). The hotspot regions of *PTEN* (exons 7 and 8) and *AKT1* (exon 2) were covered by primer pairs, which were designed and validated by the ALL package of the IRON-II (Interlaboratory Robustness of Next-Generation Sequencing) study (Table 2). Exons were amplified using the FastStart High Fidelity PCR System and GC-RICH PCR System kits (Roche Applied Science, Penzberg, Germany). Amplicons were purified with Ampure XP beads (Beckman Coulter, Krefeld, Germany) and libraries were quantified by Quant-iT PicoGreen dsDNA Reagent (Invitrogen, Carlsbad, CA, USA). Deep sequencing was performed on a Roche FLX GS Junior (454-Life Sciences, Branford, CT, USA) according to the manufacturer’s instructions. The minimum read depth threshold per amplicon per sample was set to 500x. Sanger sequencing was used to confirm the variations, and low-level variants (variant calling was <20%) were re-sequenced by using a different MID. After the data quality assessment, variant detection analyses were done by AVA software (GS Amplicon Variant Analyzer software version 2.5.3, Roche Applied Science). The in silico prediction tools MutationTaster [17] and SIFT [18] were used to evaluate the functional effects of identified variants in *PTEN* (NM_000314.4) and *AKT1* (NM_005163.2).

## Results

A total of 50 childhood T-ALL patients were screened for hotspot regions of *PTEN* and *AKT1 *by targeted deep sequencing. All detected variations are listed in Table 3. A total of five *PTEN *variations were found in three of the 50 T-ALL cases (6%) and all the variations occurred in exon 7, truncating PTEN in the C2-domain.

A nonsense c.781C>T, p.Q261* (rs730882131) pathogenic variant was found in one patient (P#7) with a low frequency (2.1%), and this somatic variation was evaluated as a small background clone without any clinical significance. P#7 is a 3.5-year-old boy who was classified in the medium-risk group (MRG), a responder to induction therapy who was followed for 27 months.

**Figure 1:**
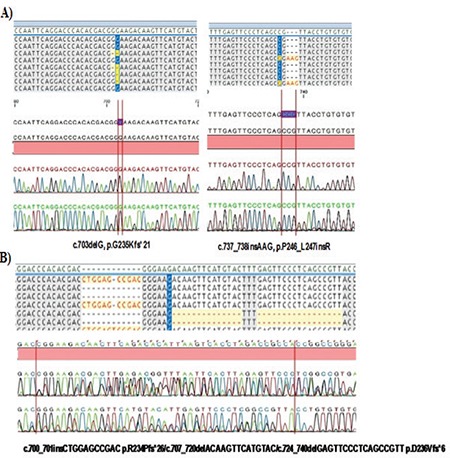
Novel variants including insertions and deletions were detected in two T-ALL cases.

Three novel variants including insertions and deletions were detected in two T-ALL cases. One patient (P#48) had two different mutant clones for *PTEN*; the first clone carried c.700_701insCTGGAGCCGAC p.R234Pfs*26 with 40% frequency and the second clone harbored c.707_720delACAAGTTCATGTAC and c.724_740delGAGTTCCCTCAGCCGTT deletions that cause p.D236Vfs*6 with 16% frequency, which are classified as “deleterious” by SIFT. The deletion area was able to be detected by conventional sequencing; however, it was not possible to distinguish the clones (Figure 1B). P#48 is a 12-year-old girl who had high white blood cell count at diagnosis (170x10^9^/L) with lymphadenopathy, splenomegaly, and hepatomegaly; she was a responder to induction therapy and has been followed in remission for 90 months.

One patient (P#27) also had two variations in the *PTEN* gene: a likely pathogenic deletion c.703delG, p.G235Kfs*21 with 10% frequency and a novel insertion c.737_738insAAG, p.P246_L247insR with 4.6% frequency (Figure 1A). She is 7 years old and classified in the MRG, a responder to induction therapy. She presented with lymphadenopathy, splenomegaly, hepatomegaly, and mediastinum involvements. She had early relapse and has now been in remission for 80 months. Furthermore, two common intronic single-nucleotide variations, rs2494749 (8%) and rs2494748 (6%), were found in the *AKT1 *gene. However, none of the patients carried the diseased-linked variation in exon 2 of the *AKT1 *gene.

*NOTCH1/FBXW7* mutation data were available for 24 of the patients [19]. None of the patients who had *NOTCH1/FBXW7 *variations carried *PTEN *or *AKT1* mutations for the respective exons. Furthermore, patients who had *PTEN* or* AKT1* variations did not carry t(9;22), t(12;21), or t(4;11).

## Discussion

PTEN has an important role in the proliferation and survival of T-cell progenitors, and its loss may sustain leukemic T-cell viability in T-ALL [20]. *PTEN* function is often inactivated by different mechanisms such as mutations, epigenetic alterations, gene silencing, and post-translational modifications in cancers where it can be associated with reduced chemotherapy response and poor prognosis [21,22].

The frequency of *PTEN* variation was previously reported as 5%-27% in different studies of T-ALL patients. Different methodologies, numbers of analyzed cases, and whole exome or hot spot region examinations may explain this diversity. In our study, exon 7 and exon 8, which are the hot spot regions for *PTEN* gene variations, were screened with targeted amplicon sequencing. Three patients had *PTEN* mutations in our cohort; on the other hand, two of the patients harbored multiple *PTEN* mutant clones that we were able to distinguish by deep sequencing. Furthermore, two patients showed low-level *PTEN* variations; we may consider that *PTEN* mutations were not the first to be hit for the oncogenic behavior in these T-ALL patients. In common with other studies, all the mutations were located in exon 7 and two novel frameshift mutations were detected in one patient, predicted to cause truncated protein. Truncating mutations located within the first eight exons of the *PTEN *gene lead to mono-allelic expression by nonsense mediated decay [23]. Furthermore, a nonsense* PTEN *variation was found in a T-ALL patient that resulted in the loss of PTEN protein levels [10].

All the patients with mutations for PTEN achieved remission after induction therapy and one patient developed early relapse. Furthermore, all patients were alive during the follow-up. PTEN is implicated in regulating downstream effects of NOTCH1 signaling such as proliferation and survival of T-cell progenitors. *PTEN* mutations were also suggested to be secondary mutations following *NOTCH1*-activating mutations, rendering cells insensitive to γ-secretase inhibitors. On the other hand, other studies suggested that *NOTCH1*-activating mutations and *PTEN *mutations were two different hits in different T-ALL subgroups [21,24]. Patients with *PTEN* mutations were particularly associated with the* TAL-1*-expressing group in T-ALL cases. In our cohort, 30% of T-ALL patients harbored *NOTCH1/FBXW7 *mutations and none of the *PTEN* mutant samples carried *NOTCH1/FBXW7 *aberrations [19].

Previous studies have reported controversial prognostic effects of *PTEN* variations in childhood T-ALL [11,13,25]. In the BFM (n=301) and GBTL1 ALL-99 (Brazilian) (n=62) pediatric ALL cohort studies, it was shown that in the absence of *NOTCH1 *mutations *PTEN* gene variations were associated with poor prognosis, while the DCOG/COALL (German) (n=146) cohort study reported *PTEN* variations as independent high-risk factors for relapse [10]. However, the UKALL2003 (n=145) pediatric cohort could not find any association between *PTEN* variations and clinical findings [11]. An Italian study group examined 257 children with T-ALL treated with AIEOP-BFM protocols. They found an association between increased risk of relapse and *PTEN *mutations in pediatric T-ALL [26]. In another study, Szarzynska-Zawadzka et al. [27] screened 162 patients with T-ALL for *PTEN *aberrations (mutations, copy number variations, and deletions) and found that *PTEN* deletions were more common than mutations (16% vs. 9%) in the patients. Additionally, bi-allelic inactivation of PTEN (co-occurrence of deletions and mutations) was detected in 8% of patients. PTEN deletions were associated with worse survival and increased risk of relapse. However, *PTEN* mutations were associated with poor survival but not with relapse. These findings suggest the existence of multiple leukemic sub-clones displaying various PTEN anomalies, with each of these subsets possibly having different biological and clinical features. Detailed analysis of the type of genetic anomaly would be useful to refine risk stratification based on PTEN status.

### Study Limitations

This study has some limitations. The number of patients in the study is limited and the patients had only been screened for the hot spot regions of the genes, although variation frequencies are similar to those of other studies.

## Conclusion

PTEN tumor suppressor gene inactivation is a frequent event in T-ALL, but its effect on patient therapy response is debatable. Herein, only a small proportion of T-ALL patients had* PTEN *and *AKT1 *variations. Therefore, it is not possible to reach a meaningful conclusion about the prognostic value of *PTEN* mutations in T-ALL. In our cohort, screening for* PTEN *abnormalities at diagnosis did not add further information about patients’ risk groups. However, the *PTEN *genotype may serve as a potential biomarker for targeted therapy in later perspective studies. Furthermore, *PTEN *mutations are not the only aberrations that contribute to the loss of PTEN protein in T-ALL patient samples. Other PTEN aberrations (copy number variations, deletions), different molecular mechanisms like effective* PTEN*-splicing, long noncoding RNAs, and epigenetic modulations that also lead to *PTEN *inactivation should also be evaluated in the future. The PTEN/AKT pathway has a critical role in cell growth and survival and has become a target pathway for pharmacological studies due to its frequent activation in various types of tumors [28,29,30,31,32]. In order to identify patients who may benefit from novel developed therapeutics, it is important to characterize the molecular background of the patients.

## Figures and Tables

**Table 1:**
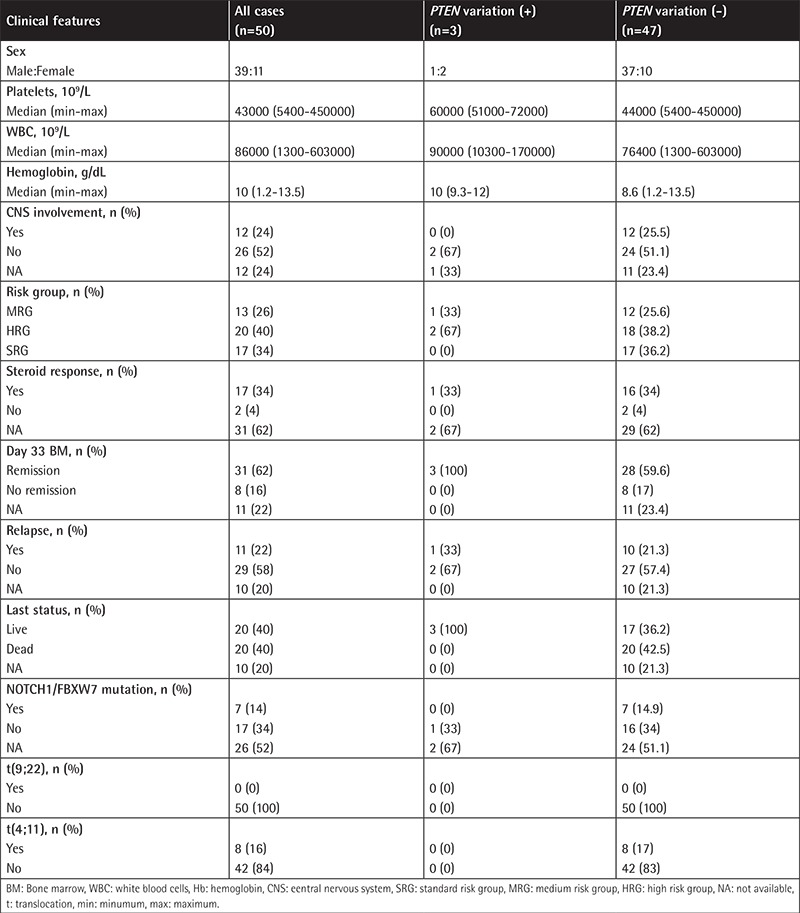
Clinical features of childhood T-ALL patients.

**Table 2:**
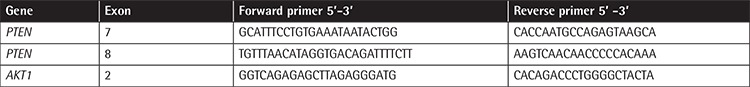
Gene-specific primer sets for deep sequencing.

**Table 3:**
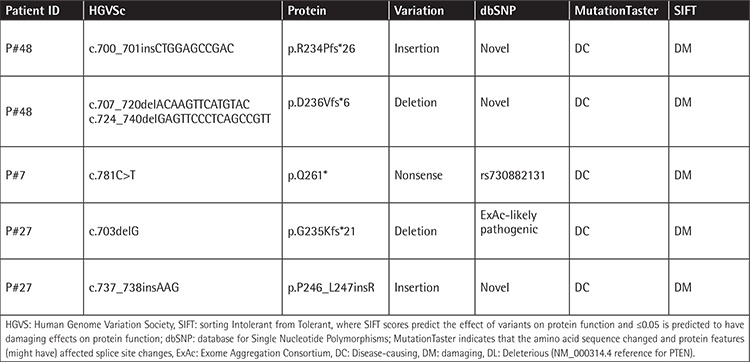
Pathogenic PTEN variations in childhood T-ALL patients.
